# Systemic and Lung Inflammation and Oxidative Stress Associated With Behavioral Changes Induced by Inhaled Paraquat Are Ameliorated by Carvacrol

**DOI:** 10.1155/2024/4049448

**Published:** 2024-08-24

**Authors:** Arghavan Memarzia, Fatemeh Amin, Amin Mokhtari-Zaer, Zohre Arab, Saeideh Saadat, Mahrokh Heydari, Zahra Ghasemi, Farzaneh Naghdi, Mahmoud Hosseini, Mohammad Hossein Boskabady

**Affiliations:** ^1^ Applied Biomedical Research Center Mashhad University of Medical Sciences, Mashhad, Iran 9177948564; ^2^ Physiology–Pharmacology Research Center Research Institute of Basic Medical Sciences Rafsanjan University of Medical Sciences, Rafsanjan, Iran; ^3^ Department of Physiology and Pharmacology School of Medicine Rafsanjan University of Medical Sciences, Rafsanjan, Iran; ^4^ Student Research Committee Torbat Jam Faculty of Medical Sciences, Torbat Jam, Iran; ^5^ Department of Basic Medical Sciences Mashhad University of Medical Sciences, Mashhad, Iran; ^6^ Department of Physiology Faculty of Medicine Mashhad University of Medical Sciences, Mashhad, Iran 9177948564; ^7^ Department of Physiology School of Medicine Zahedan University of Medical Sciences, Zahedan, Iran 9816743175; ^8^ Cutaneous Leishmaniasis Research Center Imam Reza Hospital Mashhad University of Medical Sciences, Mashhad, Iran

**Keywords:** carvacrol, learning, lung injury, memory, pioglitazone, paraquat

## Abstract

Paraquat (PQ) is an herbicide toxin that induces injury in different organs. The anti-inflammatory and antioxidant effects of carvacrol were reported previously. The effects of carvacrol and pioglitazone (Pio) alone and their combination on inhaled PQ-induced systemic and lung oxidative stress and inflammation as well as behavioral changes were examined in rats. In this study, animals were exposed to saline (control [Ctrl]) or PQ (PQ groups) aerosols. PQ-exposed animals were treated with 0.03 mg/kg/day dexamethasone (Dexa), 20 and 80 mg/kg/day carvacrol (C-L and C-H), 5 mg/kg/day Pio, and Pio+C-L for 16 days. Inhaled PQ markedly enhanced total and differential white blood cell (WBC) counts, nitric oxide (NO), and malondialdehyde (MDA) levels but decreased catalase (CAT) and superoxide dismutase (SOD) activities and thiol levels both in the bronchoalveolar lavage fluid (BALF) and blood and increased interferon-gamma (INF-*γ*) and interleukin-10 (IL-10) levels in the BALF (*p* < 0.001 for all cases) except lymphocyte count in blood which was not significantly changed. The escape latency and traveled distance were increased in the PQ group. However, the time spent in the target quadrant in the Morris water maze (MWM) test and the duration of time latency in the dark room in the shuttle box test were reduced after receiving an electrical shock (*p* < 0.05–*p* < 0.001). Inhaled PQ-induced changes were significantly improved in carvacrol, Pio, Dexa, and especially in the combination of the Pio+C-L treated groups (*p* < 0.05–*p* < 0.001). Carvacrol and Pio improved PQ-induced changes similar to Dexa, but ameliorative effects produced by combination treatments of Pio+C-L were more prominent than Pio and C-L alone, suggesting a potentiating effect for the combination of the two agents.

## 1. Introduction

Poisoning with agricultural toxins is a serious problem in today's world [1]. Paraquat (PQ, N,N′–dimethyl-4,4′–bipyridinium) is used as an herbicide and it is highly toxic to humans [2, 3]. Exposure to PQ may cause mortalities and multiorgan failure [2, 3]. The lung is the main target for PQ, where this chemical induces edema, fibrosis, inflammation, hemorrhage, and epithelial cell infiltration. The main complication of PQ poisoning that causes death is respiratory failure [4]. In addition, the induction of systemic oxidative stress and inflammation was shown due to PQ poisoning [5, 6]. PQ exposure also induces neurodegenerative diseases such as Alzheimer's and Parkinson's disease due to its effects on the oxidative system and neurodegenerative toxicity in the brain [3]. Also, exposure to PQ causes morphologic changes in the hippocampus which lead to impairment of learning and memory formation in the hippocampal neurons [7]. Although intact skin minimally absorbs PQ, once absorbed, the chemical accumulates in the lung and kidney, making these organs particularly vulnerable to PQ-induced harm. The lung susceptibility to PQ toxicity is attributed to the fact that PQ accumulates in alveolar Types I and II and Clara cells, leading to pulmonary concentrations that are 6- to 10-fold greater than those found in the bloodstream [8]. The PQ's volume of distribution fluctuates between 1.2 and 1.6 L/kg. Roughly 90% of the PQ that has been absorbed is excreted unchanged through urine within a timeframe of 12–24 h after ingestion [9]. Toxicity typically develops in the kidneys and lungs over a span of 2–6 days when smaller quantities are ingested [10].

The precise reason behind PQ toxicity remains unclear, as potential contributors may include oxidative damage, inflammation response, programmed cell death, disrupted regulation of the extracellular matrix, coagulation abnormalities, and autophagy [11]. Multiple investigations have also revealed that PQ at a dosage ranging from 0.1 to 1 mM has yielded the emergence of markers indicating oxidative stress. Subsequently, this radical undergoes a process of deoxidization, resulting in the production of a cation alongside the generation of both a superoxide anion and a hydroxyl free radical. As a consequence, these reactive species inflict harm and ultimately lead to the demise of various categories of mammalian cells, which encompass fibroblasts, lymphocytes, neuronal cells, and pulmonary epithelial cells [12–14].

Peroxisome proliferation activating receptors (PPARs) mediate metabolism pathways including *β*-oxidation of fatty acids and lipid synthesis and showed potent anti-inflammatory effects [15]. These receptors inhibit transforming growth factor (TGF) and suppress the production of inflammatory cytokines [16]. Activation of these receptors leads to regulation of cellular metabolism [17].

Carvacrol [C_6_H_3_CH_3_ (OH) (C_3_H_7_)], a monoterpenoid phenol, is a bioactive compound present in some plants, such as mint and thyme, which showed antibacterial effects [18]. Carvacrol is a constituent of several aromatic plants such as *Zataria multiflora* (*Z. multiflora*) and black cumin (*Nigella sativa*) which are frequently used in Chinese and other traditional medicines [19, 20]. Moreover, carvacrol possesses anti-inflammatory, antioxidant, and immunomodulatory effects [21].

Systemic inflammation and oxidative stress, lung edema, fibrosis, and inflammation as well as learning and memory impairment were shown following exposure to PQ. In addition, the anti-inflammatory effect of carvacrol and its protective effect on learning and memory impairment were previously demonstrated [22]. In our previous studies, the effects of *Z. multiflora* extract and carvacrol on PQ-induced systemic inflammation and oxidative markers in rats were examined by measurement of serum levels of NO_2_, malondialdehyde (MDA), IL-6, and interferon-gamma (INF-*γ*) [23]. In the present investigation, a comprehensive examination was undertaken to evaluate the impact of carvacrol, pioglitazone (Pio), and their combined administration on both systemic and pulmonary oxidative stress and inflammation, alongside the assessment of behavioral impairment provoked by the inhalation of PQ, in a rat model.

## 2. Methods

### 2.1. Study Groups

Wistar rats were housed in the Animal House, School of Medicine, Mashhad University of Medical Sciences, Mashhad (MUMS), Iran, at a standard condition outlined in our previous study [24]. The Ethics Committee of MUMS approved the study (code: 961434), and the care and use of laboratory animals were followed by national laws. In addition, the study adheres to ARRIVE reporting guidelines.

One hundred twelve male Wistar rats (200 ± 20 g) were randomly divided into 14 groups. The Ethics Committee of MUMS (code: 961434) and national statutes relating to welfare were meticulously adhered to during the animal trials. Rats were randomly divided into seven groups (*n* = 10 in each group) and used for the measurement of systemic and behavioral evaluations as follows:

1. Exposed rats to saline aerosol (control [Ctrl] group).
2. Exposed rats to 54 mg/m^3^ PQ aerosol (Sigma-Aldrich Co, China) but received no other treatments (PQ) [6, 23, 25].
3. Exposed rats to PQ and treated with 5 mg/kg/day Pio (Sigma-Aldrich Co, China) for 16 days [24, 26].
4. Exposed rats to PQ and treated with 20 and 80 mg/kg/day (C-L and C-H, respectively) carvacrol (pharmaceutical grade, Ji'AnHaiRui Natural Plant Co., China, purity 90%) [27].
5. Exposed rats to PQ and treated with Pio+C-L.
6. Exposed rats to PQ and treated with 0.03 mg/kg/day dexamethasone (Dexa) (Sigma, St. Louis, MO, Germany) [24].

**Figure 1:**
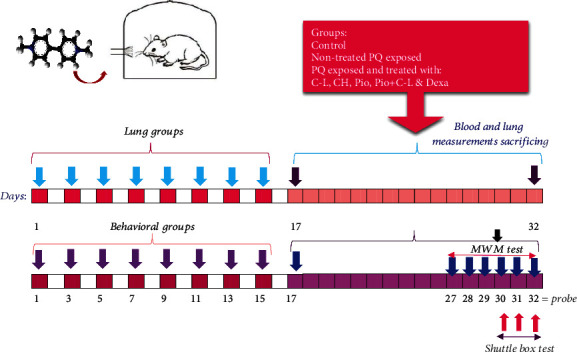
The protocol of the study for easement of systemic and lung variables (top panel) and behavioral evaluation (low panel).

Animal exposure to saline or PQ aerosol was done eight times, each time for 30 min during 16 days (Days 1, 3, 5, 7, 9, 11, 13, and 15). From Days 17 to 32, animals in the Ctrl and PQ groups were treated with saline and other groups with different agents (Figure 1). Pio was administered intraperitoneally (i.p.), but Dexa and carvacrol were administered by gavage. In order to reduce the pain caused by injury in animals, the drug acetaminophen with a dose of 50 mg/kg will be used as a solution in the drinking water of the animals.

For behavioral evaluations, Morris water maze (MWM) and shuttle tests were performed on the same day for all animals in each group. To assess the systemic and lung changes, total and differential white blood cell (WBC) and oxidative stress biomarkers in the bronchoalveolar lavage fluid (BALF) and cytokine concentration in the BALF were measured in six animals, and WBC and oxidative stress biomarkers in the blood were measured in four animals in each group randomly. Inclusion criteria included Wistar rats, aged 50–90 days, and weighing 200–220 g. Animals weighing less than 180 g and higher than 250 g were excluded from the experiment.

### 2.2. Exposure of Animals to PQ

Animals in the Ctrl group were exposed to saline aerosols, while those in the PQ groups were subjected to PQ aerosols. The experiment was repeated eight times, each lasting 30 min, on Days 1, 3, 5, 7, 9, 11, 13, and 15 (alternate days for a total of 2 weeks). As shown previously [24], the exposure to saline or PQ aerosols was performed in a hood, and the researcher used a face mask. The nebulizer (Omron CX3, Japan) generated the aerosol with a particle size of 3–5 *μ*m and an airflow rate of 8 L/min, directed into the animal's head box of 30 × 18 × 15 cm. On each occasion, a volume of 4.5 mL of saline or a solution containing PQ at a concentration of 1.33 mg/mL, purchased from Sigma-Aldrich Co in China, was introduced into the nebulizer chamber. The nebulizer's solution volume output was 0.15 mL/min, while its air output was measured at 3.7 L/min. Consequently, the PQ doses administered to the animals via their head box during the exposure period were 54 mg/m^3^. Following a duration of 30 min, the nebulizer was deactivated, and the rats were subsequently confined to the inhalation box for an additional 30 min. Subsequently, the animals were relocated to the designated animal room, as shown previously [24, 28, 29].

### 2.3. Assessment of Systemic and Lung Parameters

#### 2.3.1. Preparation of Blood and the BALF

On Day 33, the anesthetization of rats was done using ketamine (80 mg/kg) and xylazine (10 mg/kg), and the BALF was collected by injection of 1 mL of saline through a cannula inserted into the right lung. The lung was gently massaged, and saline was aspirated, which was repeated five times. Through cardiac puncture, a sample of blood (5 mL/rat) was taken and centrifuged at 2000 rpm for 10 min, as shown previously [27].

#### 2.3.2. Differential and Total WBC Determination

Using a Neubauer chamber, total WBC was counted in duplicate, and differential WBC was counted by preparing BALF or a blood smear stained with Wright–Giemsa as previously described [24].

#### 2.3.3. Oxidant and Antioxidant Biomarker Determination

Oxidant and antioxidant biomarkers such as nitric oxide (NO), MDA, thiol, catalase (CAT), and superoxide dismutase (SOD) levels were measured in the BALF or serum, as previously described [24, 30].

#### 2.3.4. Cytokine's Measurements

Using special enzyme-linked immunosorbent assay (ELISA) kits and according to the recommendation of the manufacturing technique (Karmania Pars, Kerman, Iran), levels of interleukin-10 (IL-10) and IFN-*γ* levels in the serum were evaluated by the method previously described [19].

### 2.4. Behavioral Evaluations

#### 2.4.1. MWM Test

The MWM experiment took place in a circular pool 150 cm in diameter, filled with water to a height of 50 cm, and maintained at a temperature of 25°C. The pool was divided into four quadrants (north, east, west, and south) for accuracy. A platform with a 10 cm diameter was placed 2 cm below the water's surface, hidden from the rats. Visual cues on the walls helped the rats remember the platform's location. A camera tracked the rats' swimming direction, sending data to a computer with Radian software for analysis and storage [24]. The rats were acclimated to the MWM test through a 3-day handling period. The rats were given the opportunity to move around the apparatus unrestricted for half a minute, helping them get used to their surroundings. During the initial day of the MWM exam, the rat was placed in the pool and only had a minute to locate the escape platform. Should the rat be unable to find the platform within the allocated time, the researcher stepped in to assist in directing the rat towards it. Following that, the rat was positioned on the platform for a duration of 15 s and later granted a 15-s break prior to moving on to the subsequent trial. Each rat underwent these events four times daily, with tests carried out in all quadrants every day for 4 days. During the subsequent 5 days of the experiments, the distance traveled by the rats and the time taken to locate the platform were evaluated in order to compare the performance of various groups. The rats swam through the pool after the platform was taken away on the fifth day. In this experiment, we measured and analyzed the time elapsed and distance covered in the specified quadrant [24].

#### 2.4.2. Passive Avoidance Test (PAT)

As shown previously [24], researchers utilize the PAT as a means of evaluating cognitive functions and memory in rats through the shuttle box test. The setup includes a dimly lit room and a brightly lit room connected by a small entryway. Before the test, each rat was allowed to explore both rooms for 5 min each day over 2 days to become familiar with the setup. Following this acclimation period, the training phase starts by placing the rat in a brightly lit room with a closed entryway. After 15 s, the entryway is opened, allowing the rat to enter the dimly lit room. Then, the door was closed, and the rats were subjected to a mild electrical current (2 mA for 2 s). The rats were carefully taken out of the apparatus after 30 s. Additional testing trials are conducted at 3, 24, 48, and 72-h intervals after the electrical current. [24, 31].

### 2.5. Statistical Analysis

In the present study, the sample size was chosen according to the previous studies [24, 31]. The mean ± SEM of the data are presented in the Results section and figures. The normal data distribution was assessed by the Kolmogorov–Smirnov test. The data among groups were compared through one-way analysis of variance (ANOVA) and Tukey's test. The behavioral data of the MVM test during the 5 days were compared among groups by repeated-measures ANOVA and Tukey's test. For statistical analysis, instat statistical software (GraphPad Software, Inc, La Jolla, United States) was used, and the level of *p* < 0.05 was regarded as a significance level. The relationship between the systemic and lung inflammatory and oxidative stress markers with a behavioral deficit due to inhaled PQ and carvacrol treatment was analyzed by Pearson's or Spearman's correlation analysis according to the normality test [32].

## 3. Results

### 3.1. Systemic Effects

#### 3.1.1. Serum Oxidant and Antioxidant Levels

Serum levels of CAT, SOD, and thiol were decreased, but MDA and NO levels were increased in the PQ group versus the Ctrl group (all, *p* < 0.001, Table 1). Carvacrol doses, Pio, Pio+C-L, and Dexa increased CAT, SOD, and thiol but decreased NO and MDA levels (except CAT and thiol levels in the Pio group and CAT, thiol, MDA, and NO levels in the C-L group) (*p* < 0.05–*p* < 0.001, Table 1). All oxidant and antioxidant variables except SOD were significantly more improved in the C-H group compared to the C-L group (for MDA, *p* < 0.01; and for other cases, *p* < 0.05). Also, all oxidant and antioxidant markers in Pio and CAT in the C-L groups were improved significantly less than Dexa (*p* < 0.05–*p* < 0.001, Table 1). However, in the Pio+C-L treated group, all oxidant and antioxidant markers were significantly more improved than Pio and C-L alone and NO than Dexa (*p* < 0.05–*p* < 0.001) (Table 1).

#### 3.1.2. Differential and Total WBC in Blood

Differential and total WBCs in blood were increased in the PQ group versus the Ctrl group except for lymphocyte count (all, *p* < 0.001). Both doses of carvacrol, Pio, and the combination of Pio+C-L and Dexa reduced differential and total WBC except for the effect of C-L on lymphocytes (*p* < 0.05–*p* < 0.001). In the C-H treated group, total and differential WBC were significantly more improved than C-L except for neutrophil and eosinophil counts (all, *p* < 0.05). The reduction of neutrophil and eosinophil in the treated groups with both doses of carvacrol, neutrophil in the Pio, total WBC, and monocyte counts in the C-L and Pio treated groups were markedly less than Dexa (*p* < 0.05–*p* < 0.001). However, total WBC and eosinophil counts in the Pio+C-L combination treated group were markedly more reduced than Dexa (both, *p* < 0.05) and Pio and C-L alone (*p* < 0.05–*p* < 0.01) groups (Table 2).

### 3.2. Lung Effects

#### 3.2.1. Oxide and Antioxidants in the BALF

The BALF levels of CAT, SOD, and thiol were decreased, but MDA and NO levels were increased in the PQ group versus the Ctrl group (all, *p* < 0.001). Carvacrol doses, Pio, Pio+C-L, and Dexa increased CAT, SOD activates, and thiol levels but decreased NO except in the C-L group. However, the MDA level was reduced only in the C-H, Pio+C-L, and Dexa treated groups (*p* < 0.05–*p* < 0.001). All oxidant and antioxidant variables in the BALF of the C-H group were improved more than C-L (both, *p* < 0.01). The levels of MDA and thiol in treated groups with both doses of carvacrol and Pio and the levels of CAT and NO in C-L and Pio groups were improved less than the Dexa group (*p* < 0.05–*p* < 0.001). However, in the Pio+C-L combination treated group, the NO level was improved more than Dexa (*p* < 0.01). In addition, all oxidants and antioxidants in the Pio alone group (for CAT, *p* < 0.001; and for other cases, *p* < 0.05) and NO, SOD, and thiol levels in the C-L alone group (all, *p* < 0.01) improved less than the Pio+C-L group (Table 3).

#### 3.2.2. Differential and Total WBC in the BALF

Differential and total WBCs in the BALF were increased in the PQ group versus the Ctrl group (for lymphocytes, *p* < 0.01; and for other cases, *p* < 0.001). Differential and total WBC were decreased in Dexa, C-H, Pio, and Pio+C-L combination groups, except monocytes in C-H and neutrophils in Pio groups, but treatment with C-L only reduced total WBC, neutrophil, and eosinophil counts compared to the PQ group (*p* < 0.05–*p* < 0.001). Total WBC, neutrophil, and eosinophil counts in treated groups with carvacrol doses and Pio were improved less than the Dexa group (*p* < 0.05–*p* < 0.001). However, the eosinophil count treated group with the Pio+C-L combination was improved more than Dexa (*p* < 0.001). In addition, total WBC, eosinophil, and neutrophil in the Pio+C-L combination group were improved more than C-L and Pio alone (*p* < 0.05–*p* < 0.001) (Table 4).

#### 3.2.3. Cytokines in the BALF

**Figure 2:**
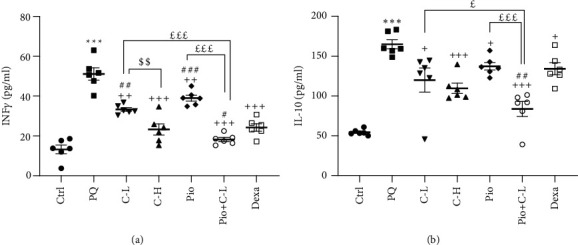
(a) The BALF levels of interferon gamma (IFN-*γ*) and (b) interleukin-10 (IL-10) of different studied groups. Statistical differences with the control group: ^∗∗∗^*p* < 0.001. Statistical differences with the PQ group: ^+^*p* < 0.05, ^++^*p* < 0.01, and ^+++^*p* < 0.001. Statistical differences with the dexamethasone group: ^#^*p* < 0.05, ^##^*p* < 0.01, and ^###^*p* < 0.001. ^$$^*p* < 0.01 C-L and C-H. Statistical differences with Pio+C-L vs. C-L and Pio alone: ^£^*p* < 0.05 and ^£££^*p* < 0.001. The data are shown as mean ± SEM (*n* = 6 in each group). One-way ANOVA followed by Tukey's multiple comparison test was used for comparisons among different groups. Ctrl: control group; PQ-54 mg/m^3^: animals exposed to 54 mg/m^3^ paraquat aerosol; Pio, C-L, C-H, Dexa, and Pio+C-L: groups exposed to PQ and treated with 5 mg/kg/day pioglitazone, 20 mg/kg/day carvacrol, 80 mg/kg/day carvacrol, 0.03 mg/kg/day dexamethasone, and pioglitazone (5 mg/kg/day) + carvacrol (20 mg/kg/day), respectively.

Flowing PQ inhalation, INF-*γ* and IL-10 levels were increased in the BALF versus the Ctrl group (both, *p* < 0.001). In all treated groups, INF-*γ* and IL-10 levels were reduced compared to the PQ group (*p* < 0.05–*p* < 0.001). The level of INF-*γ* in the C-H group was improved more than in the C-L group (*p* < 0.01). In the Pio and C-L treated groups, improvement of INF-*γ* level was less than in the Dexa group (*p* < 0.01 and *p* < 0.001 for C-L and Pio, respectively). However, in the Pio+C-L combination group, INF-*γ* and IL-10 levels were improved more than Dexa (*p* < 0.05 and *p* < 0.01 for INF-*γ* and IL-10, respectively) as well as Pio and C-L alone (for the effect of C-L on IL-10, *p* < 0.05; and for other cases, *p* < 0.001) (Figures 2(a) and 2(b)) (INF-*γ*, *F*_6,35_ = 40.87; IL-10, *F*_6,35_ = 19.73; and for both, *p* < 0.0001).

### 3.3. Behavior Effects

#### 3.3.1. MWM Results

##### 3.3.1.1. Time Latency to Reach the Stand

**Figure 3:**
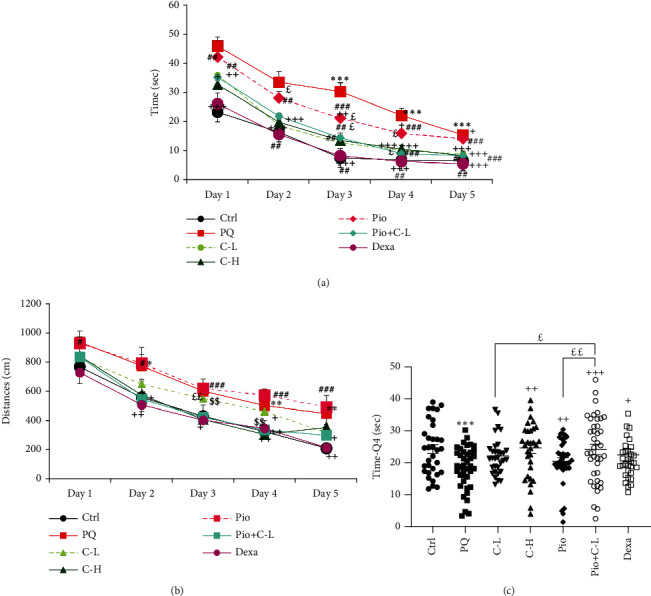
(a) Time latency, (b) traveled distance, and (c) time spent in the target quadrant on probe day measured by Morris water maze (MWM) test of different studied groups. Statistical differences with the control group: ^∗∗∗^*p* < 0.001. Statistical differences with the PQ group: ^+^*p* < 0.05, ^++^*p* < 0.01, and ^+++^*p* < 0.001. Statistical differences with the dexamethasone group: ^##^*p* < 0.01 and ^###^*p* < 0.001. Statistical differences with the combination of Pio+C-L: ^£^*p* < 0.05 and ^££^*p* < 0.01. The data are shown as mean ± SEM (*n* = 10 in each group). One-way ANOVA followed by Tukey's multiple comparison test was used for comparisons among different groups. Ctrl: control group; PQ: animals exposed to 54 mg/m^3^ paraquat aerosol; Pio, C-L, C-H, Dexa, and Pio+C-L: groups exposed to PQ and treated with 5 mg/kg/day pioglitazone, 20 mg/kg/day carvacrol, 80 mg/kg/day carvacrol, 0.03 mg/kg/day dexamethasone, and pioglitazone (5 mg/kg/day) + carvacrol (20 mg/kg/day), respectively. *F* and *p* values are shown.

The results of the MWM test showed that the time latency to reach the *stand* in the PQ-exposed group was increased in all 5 days versus the Ctrl group (all, *p* < 0.001). In all treated groups, the time latency to reach the *stand* was reduced on Days 2–5 compared to the PQ group (*p* < 0.05–*p* < 0.001). The Pio treated group took less time to reach the *stand* compared with the PQ group (*p* < 0.05–*p* < 0.001) but took more time to reach the *stand* than the Dexa group (*p* < 0.01–*p* < 0.01). The Pio+C-L group took less time to reach the *stand* across Days 2–5 compared with the Pio only group (*p* < 0.05–*p* < 0.01) (Figure 3(a) and Table 5).

##### 3.3.1.2. Traveling Distance to Reach the Stand

The traveled distance to arrive at the *stand* in the MWM examination was increased in the PQ group on all 5 days compared to the Ctrl group (*p* < 0.05–*p* < 0.01). In all treated groups, the traveled distance to attain the *stand* was reduced on Days 2–5 compared to the PQ group (*p* < 0.05–*p* < 0.001). In the C-H group, the traveling distance to reach the *stand* was less than C-L on Days 3 and 4 (*p* < 0.05–*p* < 0.01, respectively). In treated groups with Dexa and Pio+C-L, the traveling distance to reach the *stand* was less than Pio alone on all 5 days (*p* < 0.01–*p* < 0.001) (Figure 3(b) and Table 5).

##### 3.3.1.3. Probe Day

In the target quadrant in the PQ-exposed group, the time spent was decreased versus the Ctrl group on the probe day (*p* < 0.001). Treatment with C-H, C-L + Pio, and dexamethasone increased the time spent in the target quadrant on the probe day in comparison with the PQ group. In the Pio+C-L treated group, on the probe day, the time spent in the target quadrant increased higher than in the Pio and C-L alone groups (*p* < 0.05 and *p* < 0.01) (Figure 3(d), *F*_6,239_ = 2.663, *p* = 0.0161).

#### 3.3.2. Shuttle Box (Passive Avoidance) Assay

Before receiving an electrical shock, the latency to enter the darkroom was not different among the various groups. After receiving the electrical shock (3, 24, and 48 h), the time latency to enter the dark room was decreased in the PQ group versus the Ctrl group (all, *p* < 0.001).

**Figure 4:**
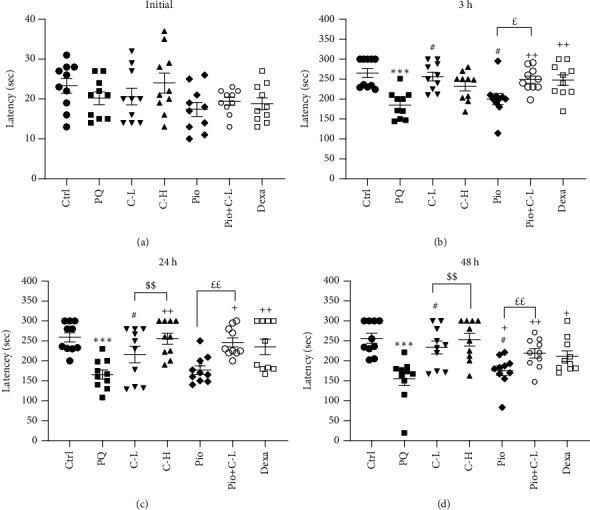
Time latency to enter the dark compartment immediately after (a) electric shock, (b) 3, (c) 24, and (d) 48 h after electric shock in passive avoidance (shuttle) test in different studied groups. Statistical differences with the control group: ^∗∗∗^*p* < 0.001. Statistical differences with the PQ group: ^+^*p* < 0.05 and ^++^*p* < 0.01. Statistical differences with the dexamethasone group: ^#^*p* < 0.05. Statistical differences with the combination of Pio+C-L: ^£^*p* < 0.05 and ^££^*p* < 0.01. ^$$^*p* < 0.01 C-L and C-H. The data are shown as mean ± SEM (*n* = 10 in each group). One-way ANOVA followed by Tukey's multiple comparison test was used for comparisons among different groups. Ctrl: control group; PQ: animals exposed to 54 mg/m^3^ paraquat aerosol; Pio, C-L, C-H, Dexa, and Pio+C-L: groups exposed to PQ and treated with 5 mg/kg/day pioglitazone, 20 mg/kg/day carvacrol, 80 mg/kg/day carvacrol, 0.03 mg/kg/day dexamethasone, and pioglitazone (5 mg/kg/day) + carvacrol (20 mg/kg/day), respectively. *F* and *p* values are shown.

In the Pio+C-L and Dexa groups, 3 h; and in those of the C-H, Pio+C-L, and Dexa groups, 24 and 48 h after an electrical shock, the time latency to enter the dark room was reduced in comparison to the PQ group (*p* < 0.05–*p* < 0.01). In the C-L group (24 and 48 h after the shock), the time latency to enter the dark room was longer than in the C-H group (both, *p* < 0.01). In the C-L and Pio treated groups (3, 24, and 48 h after shock), the time latency to enter the dark room was longer than in the Dexa group (all, *p* < 0.05). In addition, in the Pio+C-L combination treated group, 3, 24, and 48 h after the shock, the time latency to enter the dark room was increased significantly higher than in the Pio alone group (*p* < 0.05 for 3 h and *p* < 0.01 for 24 and 48 h after the electrical shock) (Figures 4(a), 4(b), 4(c), and 4(d) and Table 6).

### 3.4. The Relationship Between Behavioral Changes With Systemic and Lung Changes

There were significant positive correlations between the time spent in the target quadrant in the probe day with some antioxidant markers both in the BALF and serum but negative correlations with differential and total WBC in the BALF and blood as well as the BALF levels of INF-*γ* and IL-10 (*p* < 0.05–*p* < 0.01) (Table 1). The correlations between the latency to enter the dark room in the shuttle box test at 24 and 48 h after electrical shock with most antioxidant markers both in the BALF and serum were positive, but differential and total WBC in the BALF and blood as well as the BALF levels of INF-*γ* and IL-10 were negative (*p* < 0.05–*p* < 0.001) (Table 7).

## 4. Discussion

The influences of C, Pio, combination of Pio+C-L, and Dexa on systemic and lung injury as well as behavioral impairment induced by inhaled PQ and the association between them were evaluated in this study.

Increased differential and total WBC and oxidant but reduced antioxidant content both in the BALF and blood and IL-10 and INF-*γ* in the BALF due to inhaled PQ were shown, indicating the induction of systemic and lung oxidative stress and inflammation induced by PQ aerosol in rats. However, there was no mortality following the administration of PQ aerosol at the chosen dose during the study period.

Enhanced serum levels of oxidants and antioxidants as well as increased IL-6 levels and a reduced IFN-*γ* level and IFN-*γ*/IL-6 ratio in serum due to PQ aerosol (54 mg/m^3^) exposure in rats were reported [6]. Increased oxidant levels were positively correlated with inflammatory mediators induced by PQ administration [33]. An increased total WBC and neutrophil count in animals and humans due to PQ exposure was also reported [2, 34, 35]. Increased serum antioxidant status, lung fibrosis, TGF-1*β*, MDA level, and serum neopterin due to PQ (15 mg/kg, i.p.) were shown [36]. Longer time accumulation of PQ (six to 10 times) and its concentration in the lung compared to plasma were demonstrated [2]. Enhanced cytokine and oxidant levels, but reduced antioxidant levels in the BALF, and increased tracheal responsiveness and collagen deposition due to i.p. administration of PQ were reported [36, 37]. In 82 patients with acute PQ (10–80 mL, orally) poisoning, IL-10 expression increased, but miR-27 expression decreased. In addition, the serum expression levels of miR-27a were negatively correlated with IL-10 [38]. In 21 patients, poisoning with PQ also proinflammation cytokines such as IL-2, IL-9, IL-10, IL-2, IL-9, IL-10, and macrophage inflammatory protein-1 beta (MIP-1*β*) were elevated [39]. Therefore, the lung and systemic injury induced by PQ aerosol exposure in rats shown in this study was supported by the above studies, although PQ was administered through inhalation which was different from the previous studies.

Exposure to PQ-induced lung and systemic injury by increased production of ROS leading to lipid peroxidation, NADPH oxidation, mitochondrial toxicity, apoptosis, and nuclear factor kappa B (NF-*κ*B) activation which results in increased inflammatory mediators and cytokines was shown by PQ poisoning. This process may cause inflammatory cell infiltration, platelet aggregation, and fibrogenesis [10, 40]. The excessive production of ROS can also imbalance the redox state in cells, leading to oxidative damage including lipid peroxidation, inflammation, as well as other biologic processes, and finally, cell death [41–43].

It has been indicated that administration of PQ inhibited the activities of mitochondrial and cytoplasmic MDA and increased free radical formation and NADH autoxidation in the lungs and livers of rats [44]. Exposure to PQ also inhibited NADH, and the ubiquinone reaction (NQR) increased cytotoxicity via mitochondrial dysfunction as well as lung and blood lipid peroxidation [45]. Previous studies have shown that PQ increased intracellular ROS by inhibiting protein kinase C delta (PKC*δ*) or extracellular signal-regulated kinases (ERK1/2) and suppressing the translocation of p67phox in BV-2 cells [46]. Therefore, PQ exposure results in multiorgan injury through oxidative stress and inflammation processes which is supported by the results of this study, indicating PQ-induced lung and systemic oxidative stress and inflammation.

The effect of PQ on human health was also shown previously. The lethal dose of PQ in humans when administered orally was estimated at 35 mg/kg [47]. In addition, it was shown that exposure to inhaled PQ in closed spaces (i.e., greenhouses) caused fatal pulmonary disease [48]. Ingestion of 103 mg/kg of PQ also resulted in the deaths of 20 subjects out of 26 patients [49].

The current study showed evidence of cognitive decline and avoidance issues resulting from inhaled PQ. It was demonstrated that PQ induces both Parkinson's and Alzheimer's disease [2, 50]. During the course of 28 days, oral ingestion of PQ resulted in the formation of abnormal cells in the hippocampus along with apoptotic or necrotic changes and a decrease in Nissl bodies, as well as elevated levels of MDA, ROS, and 8-OHdG in mitochondria [7, 51]. In this research, findings reinforce the connection between inhaling PQ and experiencing learning and memory difficulties. PQ easily crosses the blood–brain barrier (BBB), leading to lipid oxidation and damage in brain tissue, as well as suppressing the release of catecholamines in the brain and neurons. [52].

Treatment with Pio, C doses, Pio+C-L, and Dexa doses of exposed animals to inhaled PQ reduced differential and total WBC counts, MDA, NO, IL-10, and INF-*γ* levels but increased SOD and CAT activities, and total thiol content in the BALF and blood. However, in the Pio+C-L group, the improvement in most measured parameters was higher than in Pio or C-L alone.

In a previous study, pretreatment with 10 mg/kg Pio inhibited myeloperoxidase activity after aspirin administration [53]. Activated PPARs and inhibition of cyclooxygenase-2 (COX-2) were shown by C. Pio also increased IL-4 but decreased TNF-*α*, IFN-*γ*, and IL-6 productions [54]. Pio treatment in lipopolysaccharide (LPS)-stimulated astrocytes inhibits NO and proinflammatory cytokines but enhances the levels of IL-4 and IL-10 [55, 56]. The antioxidant, immunomodulatory, and anti-inflammatory properties of carvacrol were well described [57]. These studies supported the antioxidant and anti-inflammatory properties of Pio and C in systemic and lung injury induced by PQ aerosols observed in this study.

The learning deficit induced by PQ was improved by Pio, C, Pio+C-L, and Dexa after treatments. These interventions reduced the time taken and distance traveled to reach the stand but increased the time spent in the target quadrant during probe tests.

Juvenile hypothyroid rats exhibited cognitive impairment and increased oxidative stress in brain tissue due to Pio administration [58]. Treatment with 10 mg/kg Pio was shown to decrease hippocampal neurodegeneration, decrease hippocampal microglia expression, reduce *β*-amyloid oligomer deposition, and improve cognition [59]. At a dosage of 30 mg/kg, Pio enhanced the growth of new neurons in the hippocampus and displayed a protective impact on rats with lesions caused by 6-hydroxydopamine, potentially as a result of its antidepressant properties [60]. By preventing behavioral impairment induced by LPS in rats, Pio demonstrated its ability to improve levels of both BDNF and IL-10, along with reducing oxidative stress markers [61].

C administration in LPS-challenged rats was effective in preventing cognitive decline, reducing brain oxidative stress, and mitigating inflammation [22]. Hence, these studies demonstrated the impact of Pio and C on cognitive impairment.

The remarkable discovery from this study was the superior outcomes of treatment with a combination of Pio+C-L on lung and systemic inflammation, oxidative stress, and learning and memory issues induced by inhaled PQ over using just C-L or Pio separately. The results could be pointing towards PPAR-*γ*s's potential impact on the observed effects of C in this research.

In fact, the inhibitory effect of C on COX-2 and activation of PPAR was reported which supports this mechanism for C [56]. However, to confirm this suggestion, using PPAR-*γ* antagonists, the effect of C should be examined.

Dexa also demonstrated soothing effects on both systemic and lung inflammation and oxidative stress, as well as cognitive impairments caused by inhaling PQ, mirroring the impacts of C, Pio, and particularly the combination of Pio+C-L. C appears to influence the injury of both the lungs and the overall body by utilizing its antioxidant and anti-inflammatory attributes. A significant correlation between the inflammatory and oxidative stress markers of the systemic and lung and behavioral deficits due to inhaled PQ and C treatment is the other novel finding of the present study. These relationships may indicate an association between systemic, lung, and behavioral changes induced by inhaled PQ and C treatment. However, this suggestion as well as the cause-and-effect role of the three types of changes should be examined in the future.

Therefore, this study provides novel findings regarding the induction of systemic and lung injury and learning and memory deficits due to inhaled PQ which could be important consequences of health effects in farmers exposed to this herbicide. The findings also indicated the therapeutic effects of carvacrol for PQ-induced disorders, perhaps through PPAR-*γ* receptors.

The effect of inhaled PQ and the influence of treatment with C and in combination with Pio on the GSH and its related enzymes like GPx, GST, GR, G6PDH, the oxidized glutathione level, the markers of oxidative stress LOOH, and protein carbonyls and ROS in the lung and blood which the limitations of this study should be examined in further studies.

## 5. Conclusion

The therapeutic benefits of carvacrol and Pio were evident in the improvement of lung and systemic injuries as well as learning and memory deficits induced by PQ inhalation. Notably, a potentiated effect was seen when carvacrol and Pio were used together, suggesting PPAR-*γ* receptor mediation of carvacrol's actions. An association between systemic and lung oxidative stress and inflammation with behavioral changes induced by PQ aerosol and carvacrol treatment was observed.

## Figures and Tables

**Table 1:** Serum levels of nitrite oxide (NO), malondialdehyde (MDA), superoxide dismutase (SOD) and catalase (CAT) activities, and total thiol content in different studied groups.

	**Ctrl**	**PQ**	**C-L**	**C-H**	**Pio**	**Pio+C-L**	**Dexa**	**F**	**p** ** value**
NO (*μ*M)	0.10 ± 0.001	0.15 ± 0.002^∗∗∗^	0.13 ± 0.001^++,££^	0.12 ± 0.002^++,$^	0.13 ± 0.006^+++,££^	0.11 ± 0.001^++,##^	0.11 ± 0.001^+++^	13.11	*p* < 0.0001
MDA (*μ*M)	0.71 ± 0.03	12.01 ± 1.26^∗∗∗^	6.86 ± 0.39^++,###,££^	3.84 ± 0.47^+++,$$,##^	6.006 ± 0.92^++,###,££^	1.25 ± 0.41^+++^	1.63 ± 0.1^+++^	37.66	*p* < 0.0001
SOD (U/mL)	2.31 ± 0.31	0.43 ± 0.07^∗∗∗^	1.27 ± 0.37^+^	1.27 ± 0.26^+^	0.58 ± 0.25^++^	1.76 ± 0.45	1.73 ± 0.6^+^	3.249	*p* = 0.0203
CAT (U/mL)	0.089 ± 0.005	0.017 ± 0.002^∗∗∗^	0.029 ± 0.005^#,£££^	0.084 ± 0.01^+^	0.013 ± 0.001^##,££^	0.061 ± 0.007^++^	0.057 ± 0.001^+^	14.16	*p* < 0.0001
Thiol (U/mL)	0.33 ± 0.05	0.034 ± 0.008^∗∗∗^	0.051 ± 0.006^###,£££^	0.098 ± 0.01^++,###,$^	0.051 ± 0.01^###,£££^	0.23 ± 0.03^+++^	0.29 ± 0.02^+++^	18.69	*p* < 0.0001
DFn, DFd	6, 21								

*Note:* The data are shown as mean ± SEM (*n* = 4 in each group). One-way ANOVA followed by Tukey's multiple comparison test was used for comparisons among different groups. The full statistical report including F, DFn, DFd, and exact *p* values were presented. C-H: groups exposed to PQ and treated with 80 mg/kg/day carvacrol; C-L: groups exposed to PQ and treated with 20 mg/kg/day carvacrol; Ctrl: control group; Dexa: groups exposed to PQ and treated with 0.03 mg/kg/day dexamethasone; Pio: groups exposed to PQ and treated with 5 mg/kg/day pioglitazone; Pio+C-L: groups exposed to PQ and treated with pioglitazone (5 mg/kg/day) + carvacrol (20 mg/kg/day); PQ: animals exposed to 54 mg/m^3^ paraquat aerosol.

^∗∗∗^ *p* <0.00 (statistical differences with the control group).

^+^ *p* < 0.05, ^++^*p* < 0.01, and ^+++^*p* < 0.001 (statistical differences with the PQ group).

^#^ *p* < 0.05, ^##^*p* < 0.01, and ^###^*p* < 0.001 (statistical differences with the dexamethasone group).

^$^ *p* < 0.05 and ^$$^*p* < 0.01 (statistical differences between two doses of carvacrol).

^££^ *p* < 0.01 and ^£££^*p* < 0.001 (statistical differences with the combination of the Pio+C-L treated group.

**Table 2:** Total WBC, lymphocyte, neutrophil, eosinophil, and monocyte counts in blood of different studied groups.

	**Ctrl**	**PQ**	**C-L**	**C-H**	**Pio**	**Pio+C-L**	**Dexa**	**F**	**p** ** value**
Total WBC	4.2 ± 0.75	12.07 ± 0.58^∗∗∗^	9.13 ± 0.47^++,#,££^	7.09 ± 0.30^+++,$^	9 ± 0.64^#,+,£^	5.85 ± 0.580^+++,#^	7.625 ± 0.3^+++^	21.48	*p* < 0.0001
Neutrophil	1.06 ± 0.14	7.69 ± 0.76^∗∗∗^	5.21 ± 0.67^$,££^	4.46 ± 0.33^++^	5.50 ± 0.50^£^	2.52 ± 0.28^+++^	1.55 ± 0.47	23.04	*p* < 0.0001
Eosinophil	0.022 ± 0.004	0.293 ± 0.06^∗∗∗^	0.163 ± 0.046^##,£^	0.16 ± 0.009^++,##^	0.12 ± 0.02^##,££^	0.021 ± 0.0042^+++^	0.052 ± 0.01^+++^	9.058	*p* < 0.0001
Lymphocyte	3.059 ± 0.52	3.739 ± 0.103	3.304 ± 0.24^###,£^	2.409 ± 0.21^#^	3.17 ± 0.22^££^	2.97 ± 0.22^++,#^	3.43 ± 0.79^+^	2.158	*p* = 0.0890
Monocyte	0.049 ± 0.0048	0.348 ± 0.09^∗∗∗^	0.241 ± 0.05^#^	0.082 ± 0.007^++,$^	0.18 ± 0.045^#^	0.18 ± 0.024^#^	0.08 ± 0.022^+^	5.307	*p* = 0.0018
DFn, DFd	6, 21								

*Note:* The data are shown as mean ± SEM (*n* = 4 in each group). One-way ANOVA followed by Tukey's multiple comparison test was used for comparisons among different groups. The full statistical report including F, DFn, DFd, and exact *p* values were presented. C-H: groups exposed to PQ and treated with 80 mg/kg/day carvacrol; C-L: groups exposed to PQ and treated with 20 mg/kg/day carvacrol; Ctrl: control group; Dexa: groups exposed to PQ and treated with 0.03 mg/kg/day dexamethasone; Pio: groups exposed to PQ and treated with 5 mg/kg/day pioglitazone; Pio+C-L: groups exposed to PQ and treated with pioglitazone (5 mg/kg/day) + carvacrol (20 mg/kg/day); PQ: animals exposed to 54 mg/m^3^ paraquat aerosol.

^∗∗∗^ *p* < 0.00 (statistical differences with the control group).

^+^ *p* < 0.05, ^++^*p* < 0.01, and ^+++^*p* < 0.001 (statistical differences with the PQ group).

^#^ *p* < 0.05, ^##^*p* < 0.01, and ^###^*p* < 0.001 (statistical differences with the dexamethasone group).

^$^ *p* < 0.05 and ^$$^*p* < 0.01 (statistical differences between two doses of carvacrol).

^£^ *p* < 0.05 and ^££^*p* < 0.01 (statistical differences with the combination of the Pio+C-L treated group).

**Table 3:** BALF levels of nitrite oxide (NO), malondialdehyde (MDA), superoxide dismutase (SOD) and catalase (CAT) activities, and total thiol content in different studied groups.

	**Ctrl**	**PQ**	**C-L**	**C-H**	**Pio**	**Pio+C-L**	**Dexa**	**F**	**p** ** value**
NO (*μ*M)	0.3 ± 0.038	0.91 ± 0.071∗∗∗	0.74 ± 0.067^##,££^	0.34 ± 0.043^+++,$$^	0.6 ± 0.063^#,+,£^	0.38 ± 0.095^+++^	0.31 ± 0.062^+++^	13.65	*p* < 0.0001
MDA (*μ*M)	0.27 ± 0.008	0.41 ± 0.18^∗∗∗^	0.35 ± 0.033^#^	0.29 ± 0.02^+++, $$^	0.36 ± 0.01^#^	0.29 ± 0.02^+++^	0.3 ± 0.01^++^	6.329	*p* = 0.0003
SOD (U/mL)	3.85 ± 0.5	1.68 ± 0.1^∗∗∗^	2.12 ± 0.14^+^	2.92 ± 0.24^++,$$^	2.07 ± 0.19^££^	4.23 ± 0.4^++^	3.04 ± 0.47^+^	8.193	*p* < 0.0001
CAT (U/mL)	0.051 ± 0.002	0.032 ± 0.003^∗∗∗^	0.042 ± 0.002^+,££^	0.050 ± 0.004^++^	0.033 ± 0.003^##^	0.043 ± 0.005^+++,##^	0.039 ± 0.002^+^	3.317	*p* = 0.0136
Thiol (U/mL)	0.0091 ± 0.001	0.004 ± 0.0008^∗∗∗^	0.0048 ± 0.0006^++,##^	0.009 ± 0.0024^+++,$$^	0.0079 ± 0.0012	0.0096 ± 0.0012^++^	0.0062 ± 0.002	2.478	*p* = 0.0476
DFn, DFd	6, 28								

*Note:* The data are shown as mean ± SEM (*n* = 5 in each group). One-way ANOVA followed by Tukey's multiple comparison test was used for comparisons among different groups. The full statistical report including F, DFn, DFd, and exact *p* values were presented. C-H: groups exposed to PQ and treated with 80 mg/kg/day carvacrol; C-L: groups exposed to PQ and treated with 20 mg/kg/day carvacrol; Ctrl: control group; Dexa: groups exposed to PQ and treated with 0.03 mg/kg/day dexamethasone; Pio: groups exposed to PQ and treated with 5 mg/kg/day pioglitazone; Pio+C-L: groups exposed to PQ and treated with pioglitazone (5 mg/kg/day) + carvacrol (20 mg/kg/day); PQ: animals exposed to 54 mg/m^3^ paraquat aerosol.

^∗∗∗^ *p* < 0.00 (statistical differences with the control group).

^+^ *p* < 0.05, ^++^*p* < 0.01, and ^+++^*p* < 0.001 (statistical differences the with PQ group).

^#^ *p* < 0.05, ^##^*p* < 0.01, and ^###^*p* < 0.001 (statistical differences with the dexamethasone group).

^$^ *p* < 0.05 and ^$$^*p* < 0.01 (statistical differences between two doses of carvacrol).

^££^ *p* < 0.01 and ^£££^*p* < 0.001 (statistical differences with the combination of the Pio+C-L treated group).

**Table 4:** Total WBC, lymphocyte, neutrophil, eosinophil, and monocyte counts in the BALF of different studied groups.

	**Ctrl**	**PQ**	**C-L**	**C-H**	**Pio**	**Pio+C-L**	**Dexa**	**F**	**p** ** value**
Total WBC	3.15 ± 0.23	12.3 ± 0.84^∗∗∗^	9.5 ± 0.43^###,+,£££^	8.8 ± 0.49^++,##^	4.09 ± 0.28^+++,###,££^	5.30 ± 0.34^+++^	4.99 ± 0.67^+++^	38.38	*p* < 0.0001
Neutrophil	0.86 ± 0.060	6.33 ± 0.41^∗∗∗^	4.59 ± 0.34^###,++,£££^	4.20 ± 0.29^###,++^	5.90 ± 0.25^###,£££^	1.95 ± 0.38^+++^	1.48 ± 0.32^+++^	47.85	*p* < 0.0001
Eosinophil	0.10 ± 0.01	1.09 ± 0.13^∗∗∗^	0.36 ± 0.035^+++,##,£££^	0.33 ± 0.035^+++,#^	0.50 ± 0.11^++,#,£££^	0.08 ± 0.01^+++,##^	0.21 ± 0.03^+++^	24.67	*p* < 0.0001
Lymphocyte	2.03 ± 0.25	3.52 ± 0.34^∗∗^	3.18 ± 0.77	1.76 ± 0.25^+^	2.44 ± 0.38	2.57 ± 0.18^+^	2.96 ± 0.29	2.470	*p* = 0.0425
Monocyte	0.088 ± 0.01	1.63 ± 0.24^∗∗∗^	1.21 ± 0.19	1.14 ± 0.11	0.84 ± 0.14^+^	0.33 ± 0.03^+++^	0.17 ± 0.011^+++^	18.17	*p* < 0.0001
DFn, DFd	6, 35								

*Note:* The data are shown as mean ± SEM (*n* = 6 in each group). One-way ANOVA followed by Tukey's multiple comparison test was used for comparisons among different groups. The full statistical report including F, DFn, DFd, and exact *p* values were presented. C-H: groups exposed to PQ and treated with 80 mg/kg/day carvacrol; C-L: groups exposed to PQ and treated with 20 mg/kg/day carvacrol; Ctrl: control group; Dexa: groups exposed to PQ and treated with 0.03 mg/kg/day dexamethasone; Pio: groups exposed to PQ and treated with 5 mg/kg/day pioglitazone; Pio+C-L: groups exposed to PQ and treated with pioglitazone (5 mg/kg/day) + carvacrol (20 mg/kg/day); PQ: animals exposed to 54 mg/m^3^ paraquat aerosol.

^∗∗∗^ *p* < 0.00 (statistical differences with the control group).

^+^ *p* < 0.05, ^++^*p* < 0.01, and ^+++^*p* < 0.001 (statistical differences with the PQ group).

^#^ *p* < 0.05, ^##^*p* < 0.01, and ^###^*p* < 0.001 (statistical differences with the dexamethasone group).

^$^ *p* < 0.05 and ^$$^*p* < 0.01 (statistical differences between two doses of carvacrol).

^£^ *p* < 0.05 and ^££^*p* < 0.01 (statistical differences with the combination of the Pio+C-L treated group).

**Table 5:** The full statistical report including *F*, DFn, DFd, and exact *p* values of Morris water maze test results.

	**Time latency**					**Traveled distance**				
Days	1	2	3	4	5	1	2	3	4	5
*F*	6.141	5.600	18.99	16.35	10.05	1.130	2.119	2.802	7.182	2.006
*p* value	*p* < 0.0001					*p* = 0.3449	*p* = 0.0514	*p* = 0.0116	*p* < 0.0001	*p* = 0.0653
DFn, DFd	6, 253					6, 265				

**Table 6:** The full statistical report including *F*, DFn, DFd, and exact *p* values of the results of the Shuttle box.

	**Initial**	**3 h**	**24 h**	**48 h**
*F*	1.736	6.566	6.614	6.994
*p* value	*p* = 0.1274	*p* < 0.0001	*p* < 0.0001	*p* < 0.0001
DFn, DFd	6, 63			

**Table 7:** Correlations between behavioral changes with systemic and lung changes.

**Variables**			**Morris water maze**		**Shuttle bod**			
			**Probe day**		**24 h after electrical shock**		**48 h after electrical shock**	
			**r**	**p** ** values**	**r**	**p** ** values**	**r**	**p** ** values**
Systemic	Oxidative markers	CAT	−0.445	*p* < 0.05	0.616	*p* < 0.01	0.514	*p* < 0.05
		SOD	0.498	*p* < 0.05	0.480	*p* < 0.05	0.537	*p* < 0.05
		Thiol	0.61	*p* < 0.01	0.293	NS	0.318	NS
		MDA	−0.301	NS	−0.711	*p* < 0.001	−0.735	*p* < 0.001
		NO	−0.445	NS	−0.746	*p* < 0.001	−0.773	*p* < 0.001
	WBC	Total	0.08	NS	−0.452	*p* < 0.06	−0.507	*p* < 0.01
		Neutrophil	−0.437	NS	−0.569	*p* < 0.05	−0.634	*p* < 0.001
		Lymphocyte	−0.112	NS	−0.616	*p* < 0.01	−0.648	*p* < 0.001
		Eosinophil	0.32	NS	−0.493	*p* < 0.05	−0.466	*p* < 0.05
		Monocyte	−0.598	*p* < 0.01	−0.663	*p* < 0.01	−0.663	*p* < 0.01

Lung	Oxidative markers	CAT	0.095	NS	0.486	*p* < 0.05	0.508	*p* < 0.01
		SOD	0.510	*p* < 0.05	0.369	NS	0.478	NS
		Thiol	0.547	*p* < 0.05	0.563	*p* < 0.01	0.470	*p* < 0.05
		MDA	−0.028	NS	−0.597	*p* < 0.01	−0.650	*p* < 0.001
		NO	0.189	NS	−0.621	*p* < 0.01	−0.634	*p* < 0.001
	WBC	Total	−0.061	NS	−0.563	*p* < 0.01	−0.540	*p* < 0.01
		Neutrophil	−0.411	*p* < 0.05	−0.511	*p* < 0.01	−0.497	NS
		Lymphocyte	−0.112	NS	−0.535	*p* < 0.01	−0.447	*p* < 0.05
		Eosinophil	−0.136	NS	−0.627	*p* < 0.001	−0.640	*p* < 0.001
		Monocyte	−0.42	*p* < 0.05	−0.344	NS	−0.391	NS
	Cytokines	INF-*γ*	−0.280	NS	−0.626	*p* < 0.001	−0.664	*p* < 0.001
		IL-10	0.420	NS	−0.596	*p* < 0.01	−0.580	*p* < 0.01

## Data Availability

Data are available from the corresponding author upon request.

## Nomenclature

C: carvacrol
BALF: bronchoalveolar lavage fluid
CAT: catalase
Dexa: dexamethasone
IL-6: interleukin-6
MDA: malondialdehyde
MIP-1*β*: macrophage inflammatory protein-1 beta
NO_2_: nitrogen dioxide
NQR: ubiquinone reaction
PKC*δ*: protein kinase C delta
PPARs: peroxisome proliferation activating receptors
PQ: paraquat
ROS: reactive oxygen species
WBC: white blood cells
*Z. multiflora*: *Zataria multiflora*
